# The Role of CHK1 Varies with the Status of Oestrogen-receptor and Progesterone-receptor in the Targeted Therapy for Breast Cancer

**DOI:** 10.7150/ijbs.41627

**Published:** 2020-02-21

**Authors:** Wei Xu, Minghua Huang, Jia Guo, Huiting Zhang, Depeng Wang, Tiantian Liu, Haiting Liu, Shiming Chen, Peng Gao, Kun Mu

**Affiliations:** 1 Department of Pathology, School of Basic Medical Sciences, Shandong University, Jinan, 250012, China.; 2 Department of Respiratory and Critical Care Medicine, The second affiliated Hospital of Nanchang University, Nanchang, 330006, China.; 3 Department of Pathology, Qilu Hospital, Shandong University, Jinan, 250012, China.

**Keywords:** CHK1 inhibition, breast cancer, oestrogen-receptor, progesterone-receptor, adriamycin

## Abstract

**Objective:** The therapeutic effects of the checkpoint kinase 1 (CHK1)-targeted inhibition in tumor therapy have been confirmed, but how to choose an effective application method in breast cancer with heterogeneous molecular characteristics has remained unclear.

**Methods:** We evaluated the status of CHK1 in breast cancer using the cancer genome atlas database. Chemosensitivity and single-agent antitumor activity of CHK1 inhibition were measured by drug sensitivity assay, cell proliferation assay, cell cycle and apoptosis analysis in breast cancer with different ER/PR status. And based on the conjoint transcriptome atlas analyses, the corresponding mechanism were explored.

**Results:** In ER^-^/PR^-^/HER2^-^ breast cancer, CHK1 inhibition enhanced adriamycin (ADR) chemosensitivity which was mediated by the mitotic checkpoint complex (MCC)-anaphase-promoting complex/cyclosome (APC/C)-cyclin B1 axis, Msh homeobox 2 (MSX2) and Bcl-2-like protein 11 (BIM). However, in ER^+^/PR^+^/HER2^-^ breast cancer, because of the significant suppression for centromere protein F (CENPF)-mediated transcriptional activation of CHK1 induced by ADR itself, CHK1 inhibition fails to sensitize ADR toxicity. Interestingly, CHK1 inhibition showed the single-agent antitumor activity in ER^+^/PR^+^/HER2^-^ breast cancer which was mediated by the cyclin dependent kinase inhibitor 1A (p21), kinesin family member 11 (Eg5) and cell surface death receptor (Fas).

**Conclusions:** CHK1's variable role determines the application of CHK1 inhibition in breast cancer with ER/PR heterogeneity.

## Introduction

Application of molecular targeted intervention is increasingly recognized as a useful strategy in the treatment of breast cancer. In previous studies, we confirmed that CHK1 is associated with acquired resistance to neoadjuvant chemotherapy for breast cancer 1. Recently, more and more research into tumor therapy has focused on CHK1 inhibitors in combination with chemotherapy 2, 3 and their single-agent antitumor effects 4. However, the application of CHK1 inhibitors is limited by tumor heterogeneity in breast cancer, in response to this problem, some studies have pointed out that the therapeutic effects of CHK1 inhibition are related to p53-deficiency 5. However, this is far from sufficient to determine the relationship between application of CHK1 inhibition and tumor heterogeneity.

To cope with the heterogeneity of breast cancer, the status of ER, PR and HER2 is the major criteria of the evaluation for clinical decisions 6. Therefore, we selected these three markers to explore their relationship with CHK1 inhibition. Here, published data from The Cancer Genome Atlas (TCGA) have shown the close relationship between CHK1 and ER/PR status. We confirmed that CHK1 inhibition has different effects on ER^-^/PR^-^/HER2^-^ breast cancer than on ER^+^/PR^+^/HER2^-^ breast cancer in the aspects of proliferation, apoptosis and ADR chemosensitivity. Based on conjoint transcriptome analyses, we revealed that the reason lied in CHK1's role varying with ER/PR status. In ER^-^/PR^-^/HER2^-^ breast cancer, ADR could activate CHK1 to regulate cell cycle arrest mediated by MCC-APC/C-cyclinB1 axis and apoptosis induced by MSX2 and BIM. Instead, in ER^+^/PR^+^/HER2^-^ cancer cells, due to significant suppression of CENPF-mediated transcriptional activation for CHK1 induced by ADR itself, CHK1 inhibition failed to sensitize ADR toxicity. Interestingly, CHK1 inhibition showed single-agent antitumor activity mediated by Fas, p21 and Eg5 in ER^+^/PR^+^/HER2^-^ cancer cells. In conclusion, based on the variable role of CHK1, we demonstrated an effective application strategy for CHK1 inhibition.

## Materials and Methods

### Bioinformatics analysis

In this study, we used a variety of bioinformatics analysis strategies and tools at each experimental stage. We measured CHK1 expression in breast cancer with different ER, PR and HER2 statuses and in adjacent peritumoral tissues from the TCGA and the Genotype-Tissue Expression Program (GTEx) databases using the Gene Expression Profiling Interactive Analysis (GEPIA) and UCSC Xena. We also performed survival analysis of CHK1 in breast cancer using the Kaplan Meier Plotter, with the best-performing threshold as a cutoff. Next, we downloaded genes co-expressed with CHK1, ER, PR or HER2 from the cBioPortal. To explore the mechanism by which CHK1 regulates ADR chemosensitivity in ER^-^/PR^-^/HER2^-^ breast cancer, we pioneered a conjoint transcriptome analysis of gene data sets and phenotype data sets. The gene data sets included genes co-expressed with CHK1 from published TCGA, as well as RNA sequencing (RNA-seq) data of the si-CHK1 and si-control groups for MDA-MB-231 cells. And GSE24460 and GSE116441from the Gene Expression Omnibus (GEO) were involved in phenotype data sets. In addition, GSE763 provided information about transcriptional regulation of CHK1 by ADR, and GSE31912 was used to analyze the cytotoxicity of CHK1 inhibition in ER^+^/PR^+^/HER2^-^ breast cancer. Next, we performed the analyses of gene differential expression using R software version 3.5.0, GO enrichment using DAVID Bioinformatics Resources version 6.8 and protein association networks using STRING to take aim at potential targets. Correlation analysis provided the connection between CENPF, CDC20, GMNN, TOP2A, BRCA1, MKI67 and CHK1 using cBioPortal.

### Cell lines and cell culture

We purchased human breast cancer cell lines MCF-7, T47D, MDA-MB-231 and MDA-MB-468 from the American Type Culture Collection (ATCC; Manassas, Virginia, US). Cell authentication was verified by short tandem repeat profiling. These cell lines were cultured in Dulbecco's Modified Eagle's Medium (DMEM, MCF-7 and T47D) or Leibovitz's L15 medium (MDA-MB-231 and MDA-MB-468) supplemented with 10% fetal bovine serum (FBS, Gibco BRL, Grand Island, New York, US) in a 5% CO2 atmosphere at 37° C. We induced MDA-MB-231/ADR (ADR-resistant) cells with progressive concentrations of ADR and cultured them in Leibovitz's L15 medium supplemented with 10% FBS. All cell lines were tested as free from mycoplasma contamination by the mycoplasma PCR testing and grown for no more than 20 passages in total for any experiment.

### RNA interference, plasmids and cell transfection

GenePharma (Shanghai, China) synthesized siRNAs targeting the CHK1, CENPF, CDC20, GMNN, TOP2A or BRCA1 gene and non-targeting siRNA control according to the sequence verified by Sigma-Aldrich. The pEnter-CHK1 plasmid and the mutant of Chk1 containing alanine in place of serines 317 and 345 were purchased from GenePharma. We transfected siRNA (50 nM) or plasmid into MDA-MB-231, MDA-MB-468, MDA-MB-231/ADR, MCF-7 and T47D cells using Lipofectamine 2000 (Invitrogen, Carlsbad, California, US) per manufacturer's protocol. The knockdown and overexpression efficiency of CHK1 is confirmed by RT-qPCR and Western blot at 48 h after transfection. All siRNA sequences are shown in Table S1.

### RNA extraction and real-time quantitative PCR (RT-qPCR)

Total RNA and RT-qPCR were performed as previously described 7. Primer sequences in this study are presented in Table S2.

### Western blot analysis

We added 50 μg proteins from cell lysates to sodium dodecyl sulfate polyacrylamide gel electrophoresis (SDS-PAGE) gel and transferred them to a polyvinylidene difluoride (PVDF) membrane (MilliporeSigma, Burlington, Massachusetts, US) for blotting with antibodies against CHK1(ab40866), IP10 (ab214668), Fas (ab133619) and Eg5 (ab181981; all from Abcam, Cambridge, UK); as well as with Phospho-CHK1-Ser317 (12302S), Phospho-CHK1-Ser345 (2348S) or BIM (2933T; all from Cell Signaling Technologies [CST], Danvers, Massachusetts, US). Additionally, we used anti-GAPDH (10494-2-AP), anti-BubR1 (11504-2-AP), anti-cyclin B1 (55004-1-AP), anti-UBE2S (14115-1-AP) and anti-p21 (10355-1-AP) from Proteintech (Rosemont, Illinois, US), as well as anti-MSX2 (A2017) from ABclonal (Woburn, Massachusetts, US) and anti-CENPF (DF2310) from Affinity Biotech (Cincinnati, Ohio, US), for immunoreactivity (overnight at 4° C) which was visualized with an enhanced chemiluminescence kit (MilliporeSigma).

### Drug sensitivity assay

After we exposed cells to a gradually increasing dose of Adriamycin (MB1087, Dalian Meilun Biotech, Dalian, China) for 72 h, cell viability was assessed using the Cell Counting Kit-8 (CCK-8; K1018A, PExBIO, Houston, Texas, US) assay. Specifically, we measured ADR chemosensitivity in MCF-7, T47D, MDA-MB-231, MDA-MB-468 and MDA-MB-231/ ADR cells with or without CHK1 inhibition at final concentrations of 0, 0.1, 0.5, 1, 2, 5, 10 and 20 μM. To detect the ADR resistance factor of MDA-MB-231/ ADR, we established a relative-viability curve to assess IC_50_, which was defined as the drug concentration at which cell survival dropped to 50%. The resistance factor was equal to the IC_50_ ratio between the drug-resistant and drug-sensitive groups.

### *In vitro* drug-inducing assay

ADR-resistant cell line (MDA-MB-231/ADR) was established via intermittent induction by gradually increasing concentrations of ADR: 0.01 μM, 0.02 μM, 0.05μM, 0.1μM, 0.2μM, 0.5μM and 1 μM. Duration of ADR treatment was required to be at least 72 h. Dosage and time for drug administration were optimized and adjusted so that monoclonal drug-resistant communities appeared at each stage of the induction period. This process continued until the resistance factor met the experimental requirements; i.e., about 12 months.

### Cell apoptosis assay

We collected MDA-MB-231 and MDA-MB-231/ ADR cells transfected with CHK1-siRNA-1, CHK1-siRNA-2 or siRNA control after 48 h' exposure to ADR at a concentration of 1 or 5 μM, which was consistent with the process for the ADR-free group. In addition, CHK1-siRNA-1, CHK1-siRNA-2 or siRNA control were transfected into MCF-7 cells for 48 h to assess how CHK1 inhibition affected the apoptosis rate. Harvested cells were stained with Annexin V-fluorescein isothiocyanate (FITC) and propidium iodide (PI) using an Annexin V-FITC/PI Apoptosis Detection Kit (BestBio, Shanghai, China), following both manufacturers' protocols. We then immediately analyzed the cells via flow cytometry (Cytoflex S, Beckman Coulter, California, US).

### RNA-seq

Three pairs of MDA-MB-231 cells in si-CHK1 and si-control groups were prepared for RNA-seq (Beijing Annoroad Co. Ltd). Total RNA was extracted with TRIzol® (Invitrogen, Carlsbad, CA, USA). We depleted ribosomal RNA (rRNA) from RNA samples in using Ribo-Zero Gold Kits (Illumina, US). In accordance with the protocol of New England Biolabs (NEB; Ispwich, Massachusetts, US) Next Ultra Directional RNA Library Prep Kit for Illumina, RNA libraries were established and sequenced as 150 bp paired-end reads using the HiSeq X ten. As described elsewhere 8, we filtered RNA-seq next-generation sequencing (NGS) reads to obtain clean reads for further evaluation and analysis, including quality inspection of reads according to Phred Score, comparison to the human genome reference assembly (hg19) using HiSAT2 and merger of transcripts in StringTie. We used fragments per kilobase of transcript per million mapped reads (FPKM) to assess mRNA expression. Finally, the heatmap was generated using R software with differentially expressed genes (fold change > 2 or < 0.5,* P* < 0.1).

### Cell cycle analysis

MDA-MB-231 and MDA-MB-468 cells in the ADR and ADR-free groups were fixed and stored at -20° C for testing. We also took samples for analysis from the CHK1-siRNA-1, CHK1-siRNA-2 or siRNA control groups in the MCF-7 and MDA-MB-231 cell lines. We cultured cells in medium mixed with ADR or siRNA for 48 h, after which the harvested cells were stained with PI and analyzed by flow cytometry (Cytoflex S) per manufacturers' recommendations.

### Screening for histone methylation and deacetylation

To explore whether histone methylation and deacetylation mediated regulation of CHK1 by ADR, MCF-7 cells were cultured in DMEM with 3-Deazaneplanocin A (DZNep, A1905) or trichostatin A (TSA, A8183; both APExBIO, Houston, Texas, US). Both DZNep and TSA were dissolved in dimethyl sulfoxide (DMSO), and we ensured that the volume fraction of DMSO was < 0.01% in the cell culture medium. Specifically, per manufacturer's protocol and the previous experiment, we added DZNep at a final concentration of 0.5 or 2 μM and TSA at a final concentration of 0.05 or 0.2 μM to the medium used for cultivation of MCF-7 cells 9, 10. After the molecular inhibitors had acted for 72 h, CHK1 mRNA of MCF-7 cells in the drug and the solvent control (an equal amount of DMSO) groups was measured using RT-qPCR. Next, we applied the same process to MCF-7 cells that had been exposed to ADR at a final concentration of 0.5 μM for 48 h.

### Cell proliferation assay

Following manufacturer's instructions, cell proliferation was detected using the Cell Counting Kit-8 (CCK-8) assay at 24, 48, 72 and 96 h after transfection. After incubation for 2 h at 37° C, the absorbances were read at a wavelength of 450 nm. Additionally, EdU cell proliferation assay and colony formation were performed for further confirmation as previously described 11.

### Statistical analysis

Correlations between CHK1 levels and expression levels of genes of interest were considered significant when *P* < 0.05 and Spearman's correlation coefficient (SCC) > 0.30 or < -0.30. We used the Kaplan-Meier method for survival analysis in breast cancer with different levels of CHK1 expression, and the differences in survival curves were determined by a logrank test. Moreover, Bayes's test and the negative-binomial (nbinom) test were used for, respectively, high-throughput data from microarray profiling and RNA-seq with R software. We analyzed the correlations between molecules using linear regression for the TCGA data in breast cancer. To assess IC_50_, we used nonlinear regression to fit the relative-viability curve. Significant differences in other statistical analyses were calculated using a two-tailed Student's *t* test for two groups or one-way analysis of variance (ANOVA) for three groups. Every experiment was repeated at least 3 times. We used GraphPad Prism software version 5 (GraphPad Software, Inc., San Diego, California, US) for statistical analysis, and differences with *P* values < 0.05 were considered to be statistically significant.

## Results

### CHK1, highly expressed in breast cancer, correlates with patient survival and ER/PR status

**Figure 1:**
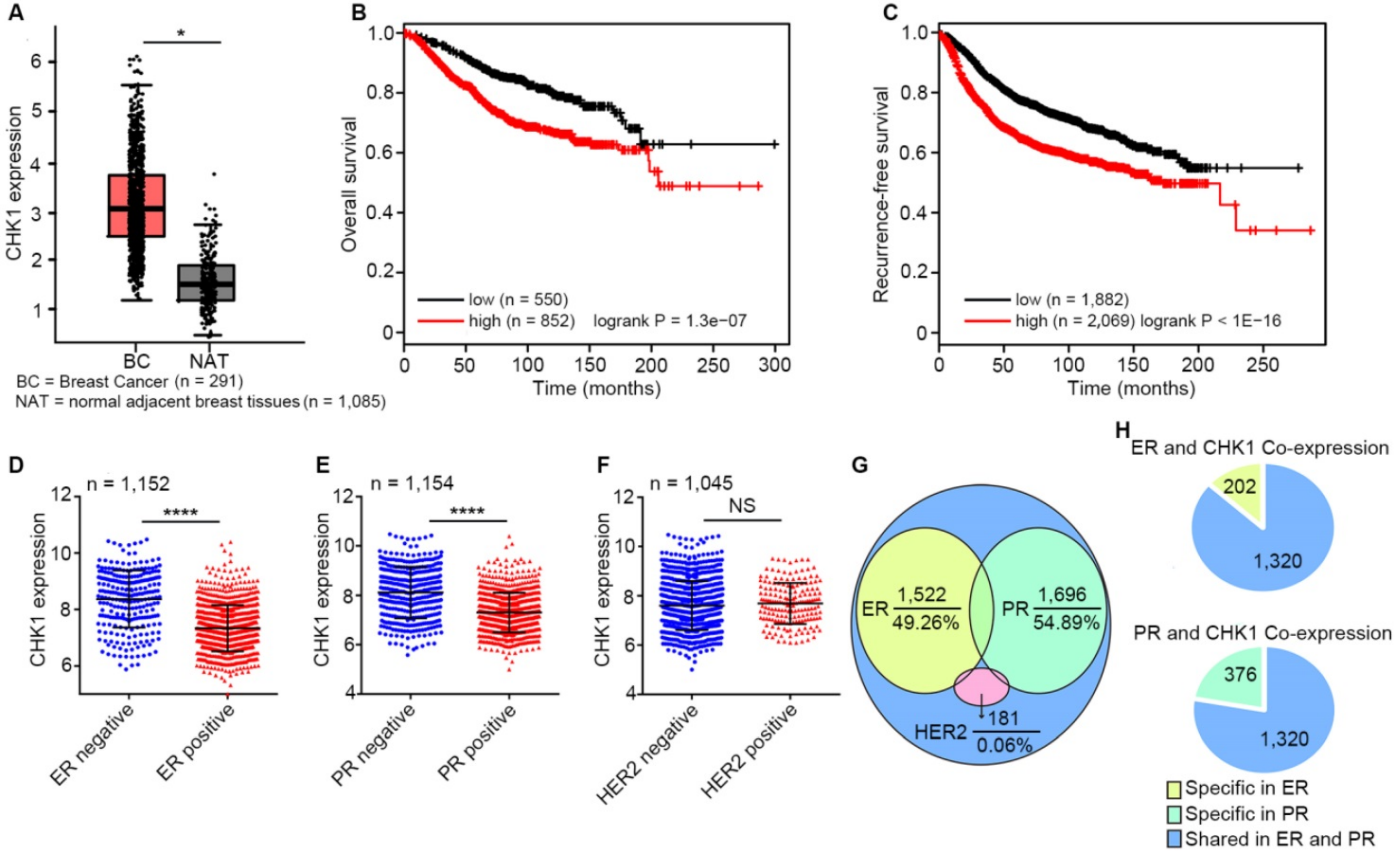
** CHK1 correlates with patient survival and ER/PR status. A** CHK1 expression in breast cancer tissues (n = 1,085) and adjacent peritumoral tissues (n = 291) from the TCGA and GTEx databases using GEPIA. **B-C** Low expression of CHK1 was associated with better overall survival (**B**, n = 1,402, logrank *P* = 1.3e-07) and recurrence-free survival (**C**, n = 3,951, logrank* P* < 1E-16) in breast cancer patients. Data were obtained from Kaplan Meier Plotter using the best-performing threshold as a cutoff. **D-F** Based on TCGA data, CHK1 expression in breast cancer patients with heterogeneous ER (**D**, n = 1,152,* P* < 0.0001), PR (**E**, n = 1,154,* P* < 0.0001) or HER2 (**F**, n = 1,045,* P* = 0.259) status. **G-H** The distribution of genes from the TCGA database co-expressed with CHK1, ER, PR or HER2 (SCC > 0.30 or < -0.30). Of genes co-expressed with CHK1, 49.26% and 54.89% were co-expressed with ER or PR, respectively, but only 0.06% were co-expressed with HER2 (**G**). Genes co-expressed with CHK1 and ER were almost identical to those co-expressed with CHK1 and PR (**H**). Data shown represent the means (± standard deviation [SD]) of three independent experiments; *P < 0.05, ****P < 0.0001; NS, not significant; logrank test (**B, C**) or Student's *t* test (**D, E, F**).

By analyzing published data from TCGA and GTEx database, we found that CHK1 expression in breast cancer tissues was significantly higher than that in adjacent peritumoral tissues (Figure 1A, n=1,376, *P* < 0.05). Moreover, survival analysis as calculated using the Kaplan-Meier Plotter showed that low levels of CHK1 predicted better overall survival (Figure 1B, n = 1,402, logrank *P* = 1.3e-07) and recurrence-free survival (Figure 1C, n = 3,951, logrank *P* < 1e-16) in breast cancer. Additionally, CHK1 was highly expressed in ER negative (Figure 1D, n = 1,152, *P* < 0.0001) or PR negative (Figure 1E, n = 1,154, *P* < 0.0001) patients, rather than correlating to HER2 status (Figure 1F, n = 1,045, *P* = 0.259). To further reveal the relationship between CHK1 and these three markers, we analyzed genes co-expressed with CHK1, ER, PR and HER2 from TCGA (SCC > 0.30 or < -0.30). We found that of the 3,090 genes co-expressed with CHK1, 49.26% and 54.89% were shared with ER co-expression or PR co-expression genes, respectively. However, the intersection of genes co-expressed with CHK1 and those co-expressed with HER2 accounted for only 0.06% (Figure 1G). Here, it is worth mentioning that genes co-expressed with CHK1 and ER were almost the same as those co-expressed with CHK1 and PR (Figure 1H). Our findings suggested a potential relationship between CHK1 and tumor heterogeneity involving ER/PR status. Therefore, we selected ER^-^/PR^-^/HER2^-^ (MDA-MB-231, MDA-MB-468) and ER^+^/PR^+^/HER2^-^ (MCF-7, T47D) human breast cancer cell lines for further research, and we found, respectively, a low-CHK1 expression and a high-CHK1 expression cell line in each type of cancer cell (Figure S1A).

### CHK1 knockdown enhances chemosensitivity to ADR in ER^-^/PR^-^/HER2^-^ breast cancer

**Figure 2:**
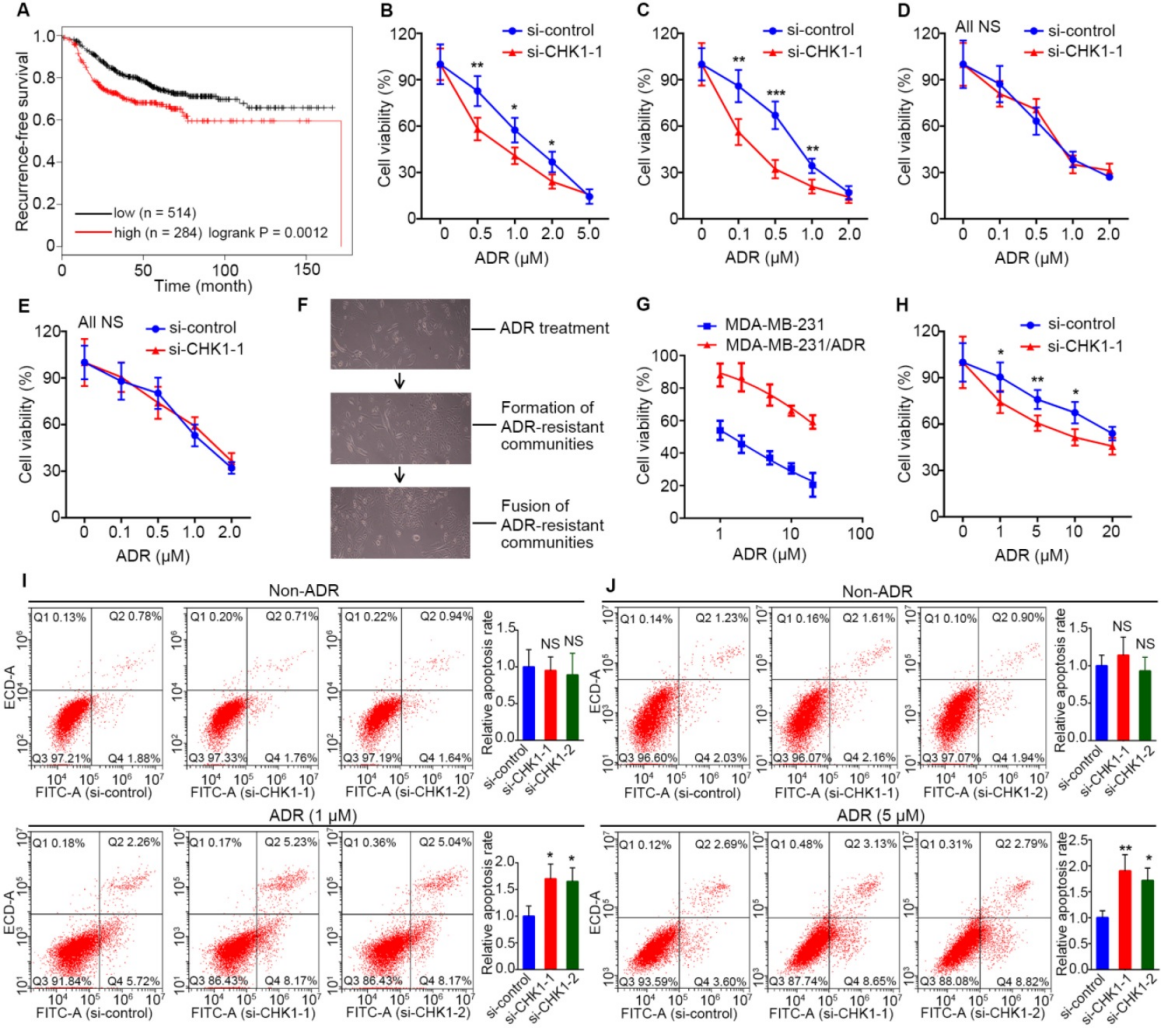
** CHK1 inhibition enhances ADR sensitivity in ER-/PR-/HER2- breast cancer. A** low level of CHK1 predicted high recurrence-free survival probability (n = 798, logrank* P* = 0.0012) in breast cancer treated with chemotherapy. Data were obtained from the Kaplan Meier Plotter using the best-performing threshold as a cutoff. **B-E** After transfection with siRNA-CHK1-1, we used a drug sensitivity assay to assess the effects of CHK1 inhibition on ADR chemosensitivity in MDA-MB-231 (**B**), MDA-MB-468 (**C**), MCF-7 (**D**) and T47D (**E**) cells. **F** The process of inducing MDA-MB-231/ADR. Each induction cycle consisted of three phases: cell death, drug-resistant community formation and integration of communities. **G** ADR resistance factor of MDA-MB-231/ADR cells. We established a relative-viability curve to assess the IC_50_s of the drug-resistant and drug-sensitive groups; the resistance factor was equal to the ratio of both groups' IC_50_s. **H** After transfection with siRNA-CHK1-1, the effects of CHK1 inhibition on ADR chemosensitivity in MDA-MB-231/ADR cells was measured using a drug sensitivity assay. **I-J** We performed an Annexin V-FITC/PI apoptosis assay to investigate the role of CHK1 in cell apoptosis in MDA-MB-231 (**I**) and MDA-MB-231/ADR (**J**) cells. Results showed that CHK1 silencing improved the ADR-induced apoptosis rate. Data shown represent the means (± SD) of three independent experiments; *P < 0.05, **P < 0.01, ***P < 0.001; NS, not significant; logrank test (**A**), nonlinear regression (**G**), Student's *t* test (**B-E, H**) or one-way ANOVA (**I, J**).

Data from Kaplan Meier Plotter showed that, for breast cancer treated with chemotherapy, high recurrence-free survival probability appeared in patients with low CHK1 levels (Figure 2A, n = 798, logrank *P* = 0.0012). To explore the effect of CHK1 on ADR chemosensitivity in breast cancer with heterogeneous ER/PR status, the siRNA targeting CHK1 or pEnter-CHK1 plasmid were transfected into the MDA-MB-231, MDA-MB-468, MCF-7 and T47D cell lines. The knockdown and overexpression efficiency of CHK1 were detected 48 h after transfection (Figure S1B-E, Figure S3A, B). In addition, using a CCK-8 assay in MDA-MB-231 and MDA-MB-468 cancer cells, we observed that CHK1 knockdown potentiated ADR-induced cytotoxicity (Figure 2B, C; Figure S2A, B), whereas the overexpression of CHK1 significantly reduced ADR cytotoxicity (Figure S3C, D). However, we found no significant difference in MCF-7 or T47D cancer cells (Figure 2D, E; Figure S2C, D; Figure S3E, F).

Furthermore, to detect the effect of CHK1 on ADR resistance in drug-resistant ER^-^/PR^-^/HER2^-^ cells, we adopted a screening method with high selectivity characterized by monoclonal-resistant communities (Figure 2F) and constructed an ADR-resistant strain of MDA-MB-231 (MDA-MB-231/ADR). CCK-8 assay results showed that the IC_50_ of resistant strains was about 24 times that of parent cells (Figure 2G). Although CHK1 was less expressed in MDA-MB-231/ADR than in MDA-MB-231 cells (Figure S1F), we still observed a significant reduction in ADR resistance for MDA-MB-231/ADR cells when CHK1 expression was suppressed (Figure 2H; Figure S1G, 2E). Consistent with the above, using flow cytometry, we found that silencing CHK1 improved the ADR-induced apoptosis rates of MDA-MB-231 and MDA-MB-231/ADR cells (Figure 2I, J).

### Activation of CHK1 by ADR depends on ER/PR status

**Figure 3:**
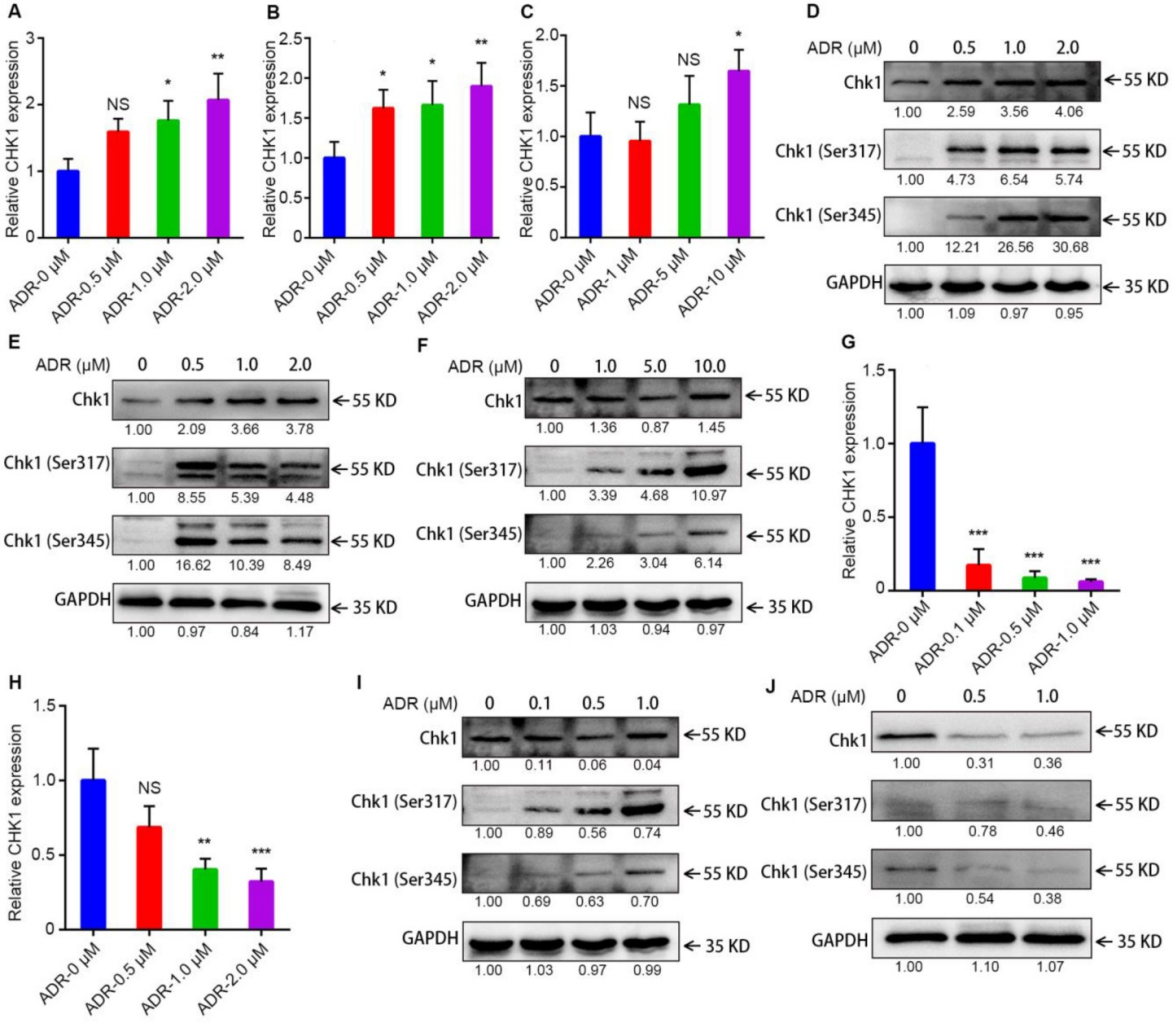
** Activation of CHK1 by ADR depends on ER/PR status. A-C, G-H** The mRNA level of CHK1 detected by RT-qPCR in MDA-MB-231 (**A**), MDA-MB-468 (**B**), MDA-MB-231/ADR (**C**), MCF-7 (**G**) and T47D (**H**) cells, with or without ADR (48 h; 0.5, 1, 2, 5 and 10 μM). **D-F, I-J** Protein levels of CHK1 in MDA-MB-231 (**D**), MDA-MB-468 (**E**), MDA-MB-231/ADR (**F**), MCF-7 (**I**) and T47D (**J**) cells, with or without ADR (48 h; 0.5, 1, 2, 5 and 10 μM), detected by Western blot. Data shown represent the means (± SD) of three independent experiments; *P < 0.05, **P < 0.01, ***P < 0.001; NS, not significant; one-way ANOVA (**A-C, G-H**).

To learn why the sensitization effect of CHK1 inhibition on ADR toxicity differed between ER^-^/PR^-^/HER2^-^ and ER^+^/PR^+^/HER2^-^ cancer cells, we measured levels of mRNA, protein and chemical modifications for CHK1 using RT-qPCR and Western blot. After activated, Chk1 is mainly phosphorylated at Ser-317 and Ser-345 12-14. The results indicated that in MDA-MB-231, MDA-MB-468 and MDA-MB-231/ADR cells, CHK1 mRNA and protein, Chk1-Ser-317 and Chk1-Ser-345 all had upward trends, especially Phospho-Chk1 (Figure 3A-F); this was the opposite trend from MCF-7 and T47D (Figure 3G-J). Moreover, compared with wild-type Chk1, the mutant of Chk1 containing alanine in place of serines 317 and 345 was poorly activated by ADR treatment and had no significant effect on ADR toxicity in MDA-MB-231 and MDA-MB-468 cells (Figure S3C, D). The above results indicated that, it was ADR's transcriptional-level regulation of CHK1 that changed CHK1's role in ER^-^/PR^-^/HER2^-^ and ER^+^/PR^+^/HER2^-^ cancer cells.

### BubR1, UBE2S, cyclin B1, MSX2 and BIM act the downstream of CHK1 to defend against ADR in ER^-^/PR^-^/HER2^-^ breast cancer

**Figure 4:**
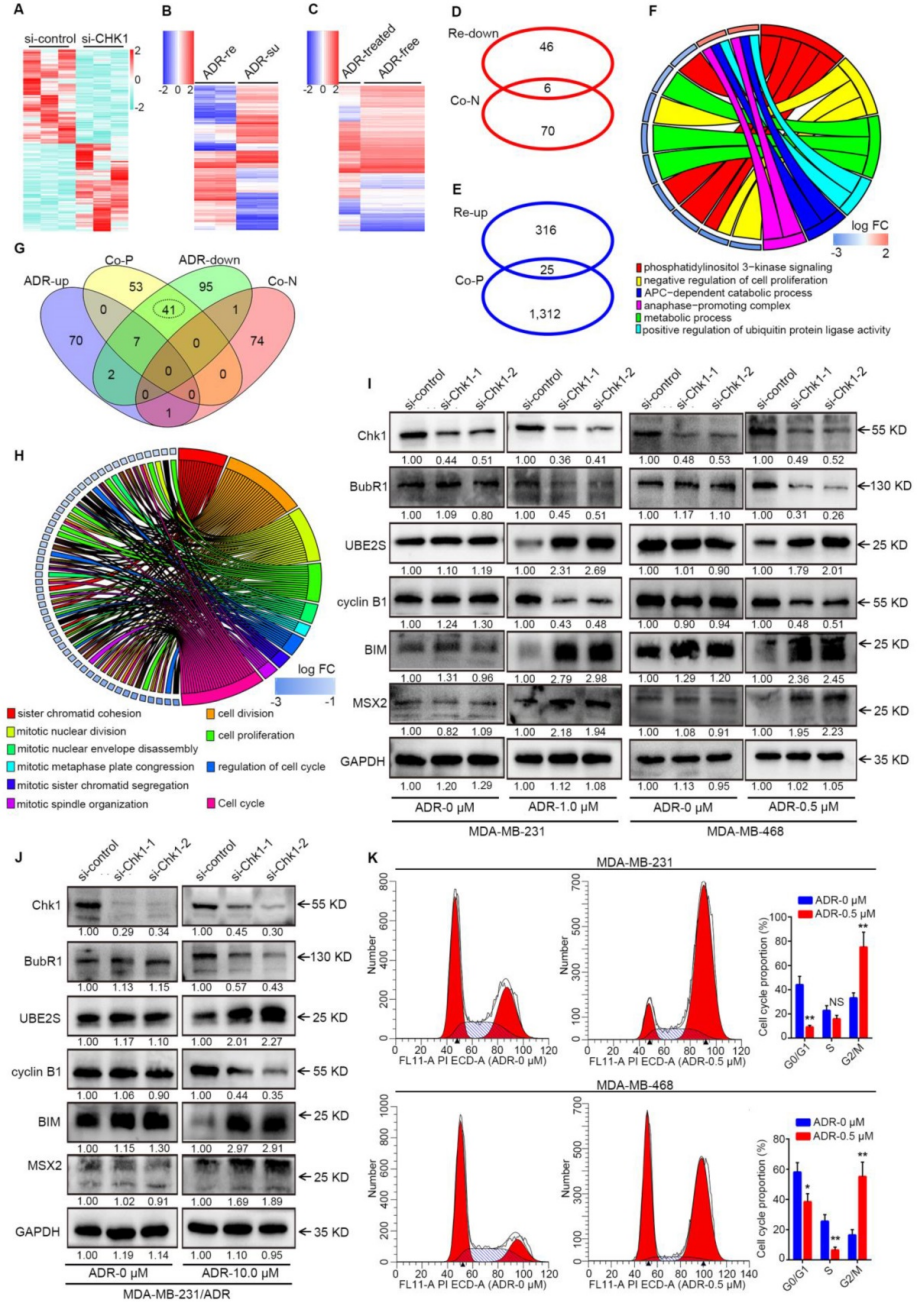
** The interactions between ADR and CHK1. A-C** Heatmap of CHK1-related data sets or phenotype data sets. Differentially expressed mRNAs (fold change > 2 or < 0.5,* P* < 0.10) examined by HiSeq X Ten sequencer in 3 pairs of si-CHK1 and si-control groups for MDA-MB-231 were involved in Figure 4A. From GSE24460 data, we analyzed differentially expressed genes (fold change > 4 or < 0.25, *q* < 0.01) between ADR-resistant (ADR-re) and ADR-susceptible (ADR-su) strains (**B**). We used variance analysis between the ADR-treated group and the drug-free group in MDA-MB-231 cells (fold change > 2 or < 0.5, *q* < 0.05) from GSE116441 data to create a heatmap (**C**). **D** After GO enrichment analysis of genes negatively associated with CHK1 (Co-N) in breast cancer from TCGA data and genes downregulated in ADR-resistant (Re-down) cells from GSE24460 data, the two groups shared 6 GO terms. **E** Intersection of genes positively associated with CHK1 (Co-P) and upregulated in ADR-resistant (Re-up) cells. **F** Based on the affiliation of genes and GO terms, the related common elements included the 6 GO terms distributed in the right half of the GO chord figure and the left 11 genes. **G** Intersection of GO terms in genes co-expressed with CHK1 and differently expressed genes from GSE116441 data including genes upregulated (ADR-up) or downregulated (ADR-down) in ADR-treated group. **H** After taking the intersection and improving the threshold of SCC, we selected 10 GO terms and 26 genes as shown in the GO chord figure. **I-J** Per verification of those selected genes by Western blot, the downstream of CHK1, including BubR1, UBE2S, cyclin B1, MSX2 and BIM, could be induced by ADR in MDA-MB-231 (**I**), MDA-MB-468 (**I**) and MDA-MB-231/ADR (**J**) cells. **K** Flow cytometry was performed to determine the effects of ADR on cell cycle distribution. A significant increase in G2/M phase and decrease in G0/G1 and S phases were observed in MDA-MB-231 and MDA-MB-468 cells. Data shown represent the means (± SD) of three independent experiments; *P < 0.05, **P < 0.01; NS, not significant; nbinom test (**A**), Bayes's test (**B, C**) or Student's *t* test (**K**). ADR-down: downregulated in adriamycin-treated group; ADR-re: adriamycin-resistant; ADR-su: adriamycin-susceptible; ADR-up: upregulated in adriamycin-treated group; Co-N: negatively associated with CHK1 in the co-expression level; Co-P: positively associated with CHK1 in the co-expression level.

In the process of mechanism research, we examined differentially expressed mRNA, defined as Group A, using a RNA-seq in si-CHK1 group and si-control groups for MDA-MB-231 cells (Figure 4A, fold change > 2 or < 0.5,* P* < 0.10). We obtained genes co-expressed with CHK1 (Group B) in breast cancer from TCGA, using SCCs greater than 0.30 or less than -0.30. Additionally, we analyzed differentially expressed genes between ADR-resistant and ADR-susceptible strains from GSE24460 data and defined them as Group C (Figure 4B, fold change > 4 or < 0.25, *q* < 0.01). Meanwhile, we obtained Group D from GSE116441 data after variance analysis between the ADR-treated group and the drug-free group in MDA-MB-231 cells (Figure 4C, fold change > 2 or < 0.5, *q* < 0.05). Next, we identified Groups A and B as gene data sets for conjoint transcriptome analyses with phenotype data sets, including Groups C and D.

For Group A, as shown in Figure S4A-B, the intersection was too minor for further analysis. Results of Western blot indicated that CHK1 inhibition significantly increased the IP10 protein with or without ADR in ER^-^/PR^-^/HER2^-^ cancer cells, compared with negative control (Figure S4C). Above all, due to a lack of genes related to the phenotype of interest, prediction for potential downstream targets of Group A was impracticable.

Previous experiments had proven that high expression of CHK1 represented an ADR-resistant phenotype. Thus, in our analysis of Groups B and C, we separately used genes negatively associated with CHK1 and genes downregulated in ADR-resistant cells for gene ontology (GO) enrichment analysis, and there were 6 GO terms in common between the 2 groups (Figure 4D). Consistently, this same process was also applied in analysis of genes positively associated with CHK1 and genes upregulated in ADR-resistant cells, except that this time we started with the intersection of genes (Figure 4E) and then did the enrichment analysis. Based on the affiliation of genes with GO terms, we were able to screen out the co-ownership parts (Figure 4F). Our analysis of Groups B and D showed a total of 50 shared GO terms, all of which related to phenotypes of interest were distributed in the 41 ones (Figure 4G). Furthermore, by taking the intersection and improving the threshold of SCC 26 genes were screened for further validation (Figure 4H). Because of the satisfactory performance of genes co-expressed with CHK1, these genes' own enrichment result was also an important reference. Additionally, among these potential targets that we had screened out, Western blot analysis showed that in MDA-MB-231, MDA-MB-468 and MDA-MB-231/ADR cells exposed to ADR for 48 h, BubR1 and cyclin B1 were downregulated, whereas UBE2S, MSX2 and BIM were upregulated, in the si-CHK1-1 and si-CHK1-2 groups (Figure 4I, J).

Our results showed that the downstream of CHK1 could be induced and expanded by ADR. Accordingly, we used flow cytometry to observe the effects of ADR on cell cycle distribution. In the MDA-MB-231 and MDA-MB-468 cell lines, ADR induced a dramatic increase in the number of cells in the G2/M phase and a decrease in the G0/G1 and S phases (Figure 4K).

### CENPF-mediated transcriptional regulation of CHK1 by ADR

**Figure 5:**
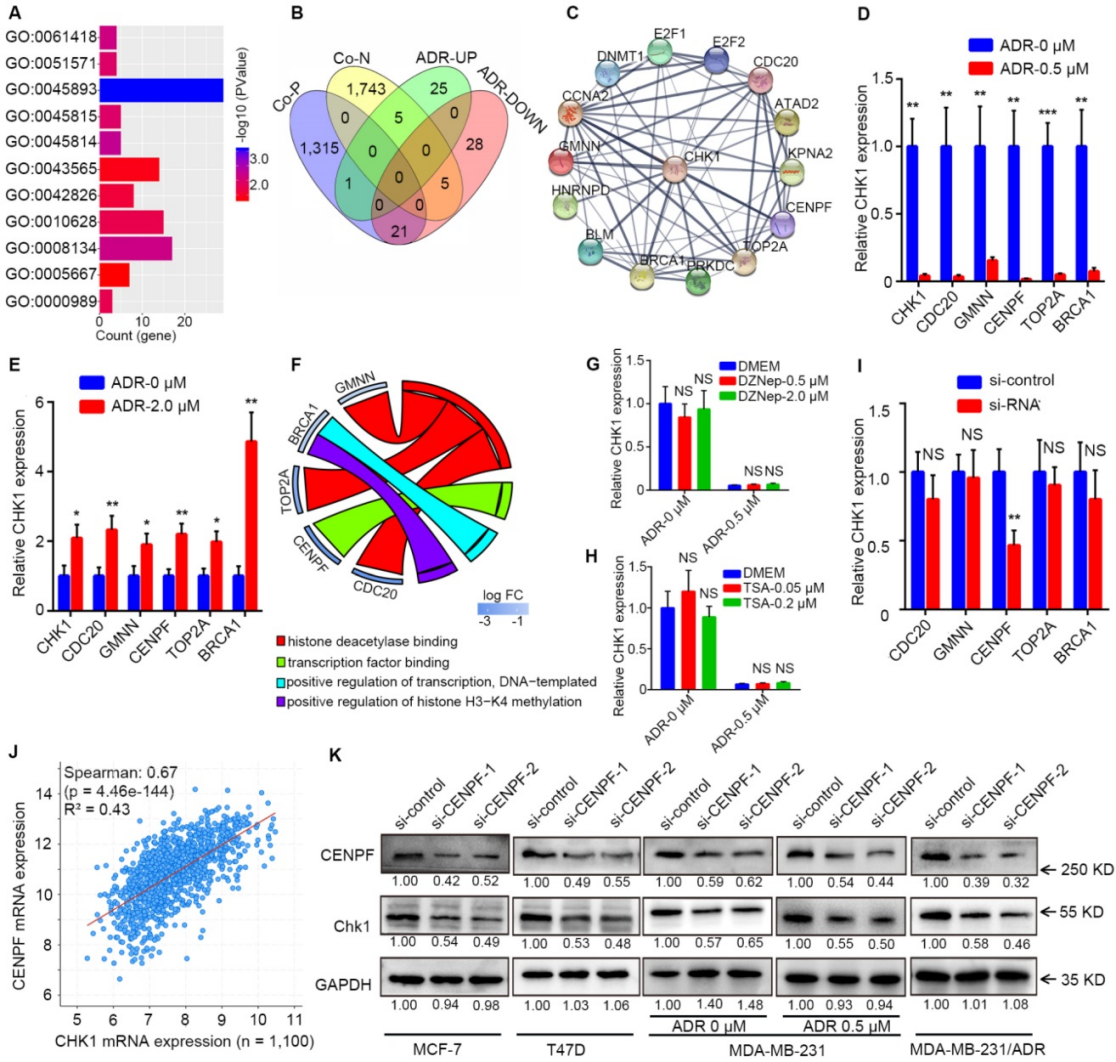
** CENPF-mediated transcriptional regulation of CHK1 by ADR. A** After GO enrichment analysis for genes differentially expressed between the ADR-treated and drug-free groups in MCF-7 cells from GSE763 data, all GO terms that could affect mRNA level were selected (*P* < 0.05). **B** Intersection of the genes in the above GO terms and genes co-expressed with CHK1 from TCGA. **C** Protein association networks of those selected genes and CHK1 were established to find targets with a comprehensive score > 0.7. **D-E** RT-qPCR showed that MCF-7 (**D**) and MDA-MB-231 (**E**) cells exposed to ADR, CDC20, GMNN, CENPF, TOP2A and BRCA1 had the same trend as CHK1. **F** GO terms associated with CENPF, CDC20, GMNN, TOP2A or BRCA1 in GSE763. **G-H** We used RT-qPCR to measure CHK1 mRNA expression in MCF-7 cells exposed to DZNep (**G**) at a final concentration of 0.5 or 2 μM or TSA (**H**) at a final concentration of 0.05 or 0.2 μM for 72 h with or without ADR at a final concentration of 0.5 μM for 48 h. **I** Using RT-qPCR, the mRNA level of CHK1 were detected in MCF-7 cells with CENPF, CDC20, GMNN, TOP2A or BRCA1 knockdown. **J** Correlation analysis of CHK1 and CENPF in breast cancer from the TCGA database using cBioPortal. **K** After transfection with CENPF-siRNA-1 or CENPF-siRNA-2, CHK1 protein was analyzed by western blot. In MCF-7, T47D, MDA-MB-231 and MDA-MB-231/ADR cells, CENPF inhibition caused significant downregulation of CHK1. Additionally, with MDA-MB-231 exposed to ADR, silencing CENPF also led to a reduction in CHK1 protein. Data shown represent the means (± SD) of three independent experiments; *P < 0.05, **P < 0.01, ***P < 0.001; NS, not significant; Student's *t* test (**D, E, I**), one-way ANOVA (**G, H**) or linear regression (**J**). ADR-UP: genes upregulated in ADR-treated group; ADR-DOWN: genes downregulated in ADR-treated group; Co-N: negatively associated with CHK1 in the co-expression level; Co-P: positively associated with CHK1 in the co-expression level.

By processing the published data from GSE763, we could perform GO enrichment analysis for the differentially expressed genes between the ADR-treated group and the drug-free group in MCF-7 to find all GO terms able to affect mRNA level, which mainly involved regulation of transcription, histone methylation and deacetylation (Figure 5A, *P* < 0.05). Genes in the above-selected GO terms with CHK1 co-expression genes from TCGA to take the intersection (Figure 5B). Next, by analyzing protein association networks for the preceding selected genes and CHK1, we obtained 14 genes with a comprehensive score of >0.70 for further validation (Figure 5C).

RT-qPCR results showed that when MCF-7 and MDA-MB-231 cells were exposed to ADR, CDC20, GMNN, CENPF, TOP2A and BRCA1 were observed to have the same trend as CHK1 (Figure 5D, E). In GO enrichment analysis, the GO terms involved in these 5 genes were transcription factor binding, positive regulation of transcription (DNA-templated), histone deacetylase binding and positive regulation of histone H3-K4 methylation (Figure 5F). DZNep and TSA are inhibitors of histone methyltransferase and deacetylase, respectively. RT-qPCR results suggested that when MCF-7 cells were exposed to DZNep or TSA at a verified effective concentration, the change of CHK1 mRNA expression was not significant, with or without ADR (Figure 5G, H). Therefore, the mode of ADR acting on CHK1 is probably not the chemical modification of histones (CDC20, GMNN or TOP2A) but transcriptional regulation (CENPF or BRCA1). Moreover, specific siRNAs for these five genes effectively downregulated the expression of their target genes in MCF-7 cells (Figure S5A). And RT-qPCR analysis revealed that only CENPF knockdown could significantly reduce the mRNA level of CHK1 in MCF-7 cells (Figure 5I). Additionally, public TCGA data indicated that CENPF was significantly positively correlated with CHK1 in breast cancer (Figure 5J, *R²* = 0.43,* P* = 4.46e-144; Figure S5B-E).

For further verification, CENPF-siRNA-1 or CENPF-siRNA-2 were transfected into MCF-7, T47D, MDA-MB-231 and MDA-MB-231/ADR cells. Western blot analysis showed that CHK1 protein levels significantly decreased in the CENPF-siRNA-1 and CENPF-siRNA-2 groups compared with negative control (Figure 5K). Moreover, when MDA-MB-231 cells were exposed to ADR, CENPF silencing also led to a reduction of CHK1 protein (Figure 5K). In other words, we demonstrated that although CENPF-CHK1 transcriptional regulation existed in breast cancer, ADR suppressed it in ER^+^/PR^+^/HER2^-^ cancer cells and enhanced it in ER^-^/PR^-^/HER2^-^ cancer cells.

### CHK1 inhibition inhibits proliferation and promotes apoptosis in ER^+^/PR^+^/HER2^-^ breast cancer

**Figure 6:**
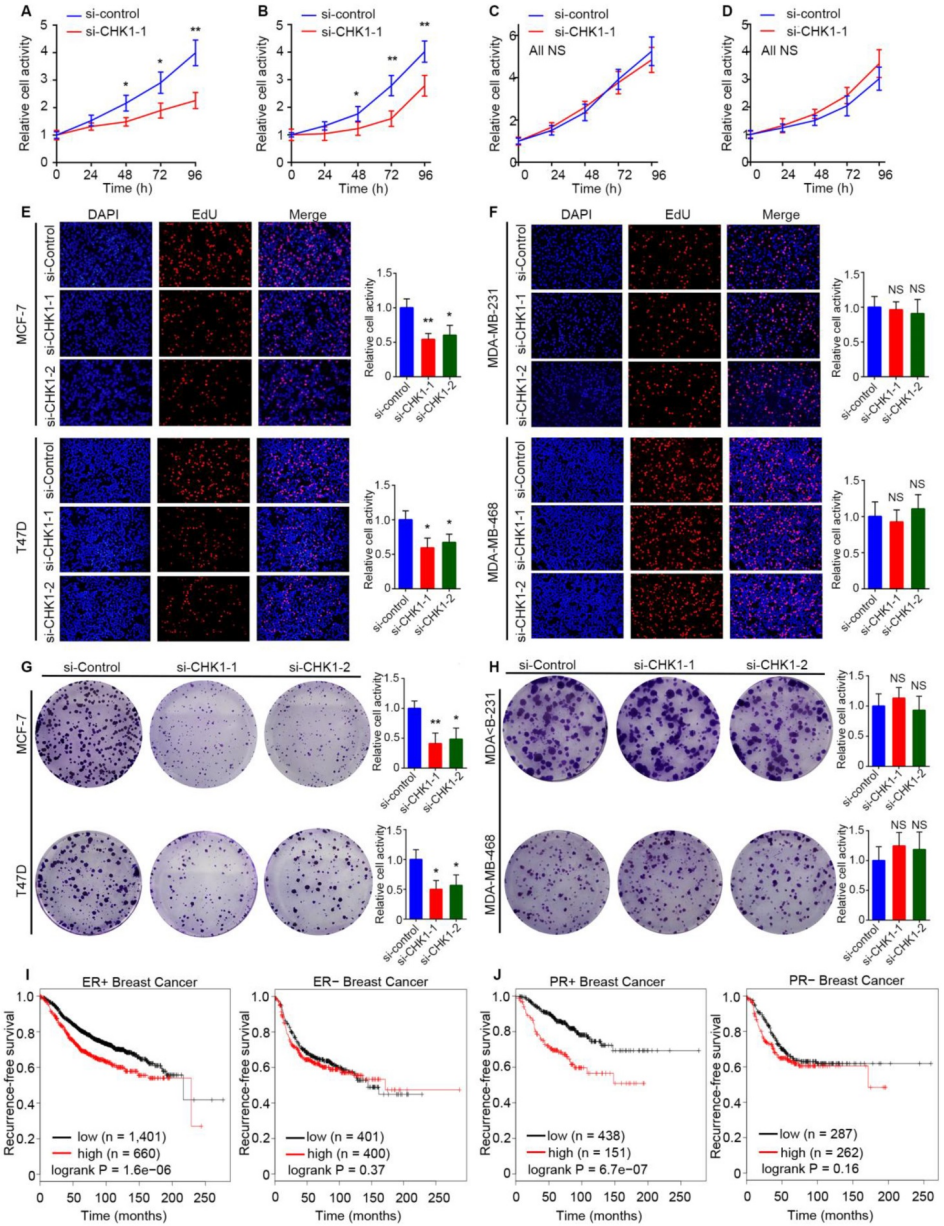
** CHK1 inhibition inhibits proliferation and promotes apoptosis in ER^+^/PR^+^/HER2^-^ breast cancer. A-D** The cell growth rates were evaluated by CCK8 assay. CHK1 knockdown (CHK1-siRNA-1) inhibited cell proliferation activities in MCF-7 (**A**) and T47D (**B**) cells but not in MDA-MB-231 (**C**) or MDA-MB-468 (**D**) cells. **E-F** Cell proliferation was determined by EdU assay in MCF-7 (**E**), T47D (**E**), MDA-MB-231 (**F**) and MDA-MB-468 (**F**) cells. **G-H** Colony formation assays were used to explore the colony formation ability of MCF-7 (**G**), T47D (**G**), MDA-MB-231 (**H**) and MDA-MB-468 (**H**) cells transfected with siRNA-CHK1-1 and siRNA-CHK1-2. **I-J** The clinical relevance of CHK1 to recurrence-free survival in breast cancer with heterogeneous ER/PR status was examined by Kaplan Meier Plotter. In ER^+^ or PR^+^ breast cancer, low levels of CHK1 predicted better recurrence-free survival; however, that was not significant in ER^-^ or PR^-^ breast cancer. Data shown represent the means (± SD) of three independent experiments; *P < 0.05, **P < 0.01; NS, not significant; Student's *t* test (**A-D**), one-way ANOVA (**E-H**) or logrank test (**I, J**).

Public TCGA data showed that MKI67, the marker of cell proliferation 15, had a significant positive correlation with CHK1 (Figure S6A, *R²* = 0.46,* P* = 1.19e-162) in breast cancer. To reveal the influence of CHK1 on cell proliferation, we used MCF-7, T47D, MDA-MB-231 and MDA-MB-468 cells as experimental objects. Interestingly, the results in ER^+^/PR^+^/HER2^-^ and ER^-^/PR^-^/HER2^-^ breast cancer were different. Both CCK-8 (Figure 6A, B; Figure S6B, C) and EdU (Figure 6E) assays showed that CHK1 inhibition dramatically weakened proliferation of MCF-7 and T47D cells. Correspondingly, a colony formation assay revealed that the number and size of colonies decreased in the CHK1-downregulation groups (Figure 6G). However, no significant correlation was found between CHK1 knockdown and cell proliferation in MDA-MB-231 or MDA-MB-468 cells (Figure 6C, D, F, H; Figure S6D, E).

To support our results with a large sample, we examined the clinical relevance of CHK1 to recurrence-free survival in breast cancer with heterogeneous ER/PR status using the Kaplan Meier Plotter. Interestingly, we found no significant difference in survival probability in patents with diverse levels of CHK1 in ER^-^ or PR^-^ breast cancer, but we did find such a difference in ER^+^ or PR^+^ patients (Figure 6I, J).

### Fas, p21 and Eg5 mediates the regulation of CHK1 on ER^+^/PR^+^/HER2^-^ cells' survival

**Figure 7:**
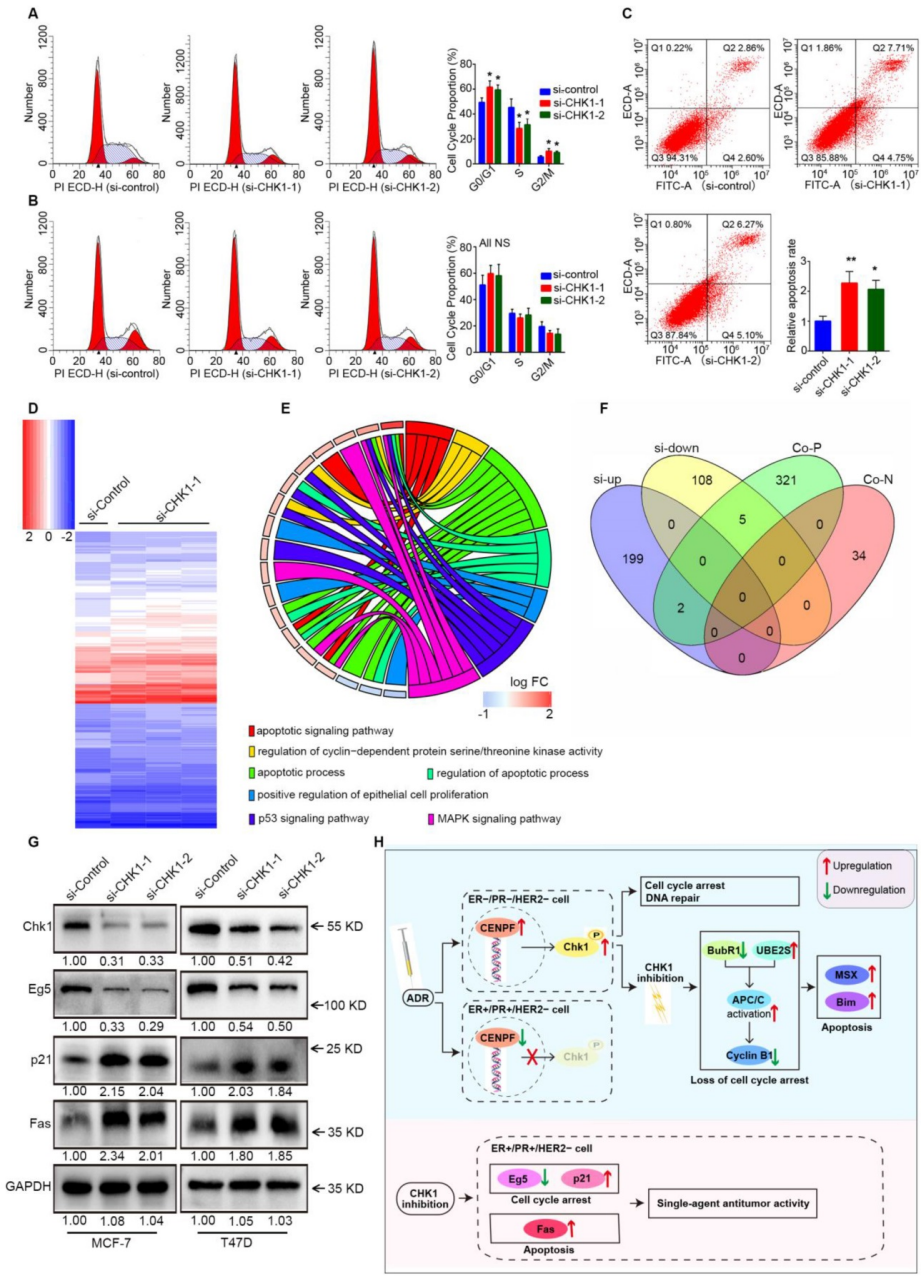
** Fas, p21 and Eg5 mediates CHK1's effect on ER^+^/PR^+^/HER2^-^ cell survival. A-B** Flow cytometry was performed to assess the effects of CHK1 suppression on cell cycle distribution. A significant S phase decrease and increase of G0/G1 and G2/M phase appeared in MCF-7 cells (**A**). No significant difference was found in MDA-MB-231 cells (**B**). **C** We also analyzed cell apoptosis by flow cytometry. We found that CHK1 inhibition induced apoptosis in MCF-7 cells. **D** Heatmap of differentially expressed genes (log2 [fold change] > 0.5 or < -0.5,* P* < 0.05) between the si-CHK1 and si-control groups in MCF-7 cells from GSE31912. **E** After GO enrichment analysis of the top 100 differentially expressed genes in GSE31912, we selected the GO terms and genes of interest as potential targets. **F** Outside of for the top 100, 7 targets were shared between differentially expressed genes from GSE31912 and genes co-expressed with CHK1. **G** Western blot results showed that with CHK1 suppressed, Fas and p21 were upregulated while Eg5 was downregulated in MCF-7 and T47D cells. **H** The role of CHK1 varies with ER/PR status in targeted therapy for breast cancer. When ADR-activated CHK1 is inhibited in ER^-^/PR^-^HER2^-^ breast cancer, the pro-apoptotic effects mediated by MSX2 and BIM are enhanced, due to the loss of cell cycle arrest mediated by the MCC-APC/C-cyclin B1 axis. However, in ER^+^/PR^+^/HER2^-^ breast cancer, the suppression of CENPF-mediated transcriptional activation for CHK1 is induced by ADR itself. The role of CHK1 inhibition is reversed, so that it shows the single-agent antitumor activity mediated by p21, Eg5 and Fas. Data shown represent the means (±- SD) of three independent experiments; *P < 0.05, **P < 0.01; NS, not significant; one-way ANOVA (**A-C**) or Bayes's test (**D**). si-up: genes upregulated in si-CHK1 group; si-down: genes downregulated in si-CHK1 group; Co-N: negatively associated with CHK1 in the co-expression level; Co-P: positively associated with CHK1 in the co-expression level.

Cell cycle distribution and apoptosis were detected by flow cytometry. Consistent with the previous results, suppression of CHK1 in MCF-7 cells dramatically decreased the number of cells in S phase and increased those in G0/G1 and G2/M phase (Figure 7A). Compared with negative control, the apoptosis rate in the CHK1-siRNA-1 and CHK1-siRNA-2 groups of MCF-7 cells was significantly higher (Figure 7C). Conversely, still no significant difference was found in the cycle distribution (Figure 7B) and apoptosis (Figure 2I) of MDA-MB-231 cells.

Downstream of CHK1 in MCF-7 cells was obtained via variation analysis of GSE31912 data (Figure 7D, log2 [fold change] > 0.5 or < -0.5, *P* < 0.05). Consistent with phenotypic differences in MCF-7 and MDA-MB-231 cells, there were no intersections in the top 200 CHK1 downstream genes ranked by multiple differences (Figure S7). Due to inconspicuous fold change caused by relatively low knockdown efficiency of CHK1, we divided differentially expressed genes into two groups for analysis: the top 100, and the remainder. We used the top 100 for GO enrichment analysis (Figure 7E) and crosslinked the remaining genes with CHK1 co-expression genes (Figure 7F) to obtain potential targets. Subsequently, Western blot results suggested that, with CHK1 suppressed, Fas and p21 were upregulated while Eg5 was downregulated in MCF-7 and T47D cells (Figure 7G).

## Discussion

In this study, a large samples data from TCGA and Kaplan Meier Plotter indicate that CHK1 is closely related to ER/PR status. As shown in Figure 7u, the role of CHK1 varies with ER/PR status in targeted therapy for breast cancer. The sensitization of CHK1 inhibition on ADR toxicity is effective in ER^-^/PR^-^/HER2^-^ cancer cells. The main mechanism involved in this process includes the loss of cell cycle arrest and the pro-apoptotic effects, mediated by BubR1, UBE2S, cyclin B1, MSX2 and BIM. However, in ER^+^/PR^+^/HER2^-^ breast cancer, the CENPF-mediated transcriptional activation for CHK1 is suppressed by ADR itself. Here, the role of CHK1 inhibition is reversed, so that it shows a single-agent antitumor activity mediated by p21, Eg5 and Fas.

According to our results, CHK1 inhibition can increase ADR chemosensitivity in ER^-^/PR^-^/HER2^-^ cells (MDA-MB-231, MDA-MB-468 and MDA-MB-231/ADR). In DNA damage response (DDR), the downstream of CHK1 are complex 16, 17, and the purpose of activated CHK1 is to buy time for the repair process, mainly by inducing G1-S and G2-M arrest 12, 18. In this study, we observed that ADR induced the extension of the downstream of CHK1mainly including BubR1, UBE2S, cyclin B1, MSX2 and BIM. It has been reported that BubR1 is involved in MCC assembly and inhibits the ubiquitination activity of APC/C in a KEN box-dependent manner 19-21. In addition, UBE2S can elongate branched conjugates that contain multiple blocks of K11-linked chains on APC/C substrates to promote recognition and degradation of substrates by APC/C 22-25. Note that the MCC-APC/C axis is the pivotal hub for spindle assembly checkpoint (SAC) to regulate the cell cycle and ensure the fidelity of chromosome segregation 26, 27. The activated SAC can inhibit ubiquitination activity of APC/C by producing MCC to prevent cyclin B1 from being degraded, which induces cell cycle arrest and drive mitotic catastrophe 28-30. In this process, if the damage is too great to be repaired, apoptosis will occur the moment the death threshold is exceeded 31. Our results indicated that in the presence of ADR, G2/M arrest was induced, and a relationship between CHK1 and the MCC-APC/C axis was established in ER^-^/PR^-^/HER2^-^ cancer cells. Specifically, with CHK1 suppressed, depletion of BubR1 and an increase in UBE2S led respectively to inhibition disorder and an enhancement of APC/C ubiquitination. Therefore, when the genome is attacked by ADR, the above changes result in the loss of cell cycle arrest and repairs. CHK1 inhibition thus enhances pro-apoptotic effects mediated by MSX2 32 and BIM 33, 34. One other point worth emphasizing in our results is that in the absence of ADR as an inductor, CHK1 does not determine cell survival.

Interestingly, when we turn our attention to ER^+^/PR^+^/HER2^-^ cancer cells, CHK1 inhibition does not act as a sensitizer for chemosensitivity of ADR. The reason lies in the fact that transcriptional regulation of CHK1 by ADR is reversed in this type of cell. At present, there have been some reports on transcriptional regulation of CHK1 35, 36. In this study, we first found that CENPF as a transcriptional activator, mediated regulation of CHK1 by ADR. According to some studies, CENPF, a nuclear-matrix component, is mainly distributed in the G2-M phase and is involved in SAC function 37. We further demonstrate that due to the significant downregulation of CENPF in ER^+^/PR^+^/HER2^-^ cancer cells, CHK1 cannot be activated to defend DDR against ADR. Therefore, this sensitization effect of CHK1 inhibition on ADR toxicity is invalid.

However, it does not mean that CHK1 inhibition loses its value in treatment for ER^+^/PR^+^/HER2^-^ cancer cells. Here, CHK1's role is reversed, which is mainly reflected by the positive effect of CHK1 inhibition on single-agent antitumor activity. Next, we found that p21, Eg5 and Fas acted as major downstream targets of CHK1 in regulating cell activity. Previous studies have confirmed that Fas, as a death receptor, triggers apoptosis by assembling the death-inducing signaling complex (DISC) 38, 39 and that p21 can inhibit cyclin-dependent kinases (CDKs) to achieve cell cycle arrest 40. As an essential component of mitotic progression, Eg5 participates in spindle assembly by driving microtubule polymerization 41. Our results thus indicated that with expression of CHK1 suppressed, cell cycle arrest was induced rather than eliminated due to an increase of p21 and loss of Eg5, and the ultimate fate of cancer cells was Fas-induced apoptosis. Based on these findings, CHK1 inhibition showed the single-agent antitumor activity in ER^+^/PR^+^/HER2^-^ cancer cells.

We did not perform *in vivo* experiments in this study, as the xenograft model constructed by Seung WooChung et al. supported our conclusions 42. Moreover, it is worth mentioning that due to our selection of different types of ER^-^/PR^-^/HER2^-^ cell lines, Christopher Bryant et al.'s results are somewhat inconsistent with ours in the role of CHK1 inhibition 43. The intrinsic properties of triple-negative breast cancer are complex and diverse 44. Although both studies used ER^-^/PR^-^/HER2^-^ cells, the focus of our study, ER/PR status, is not the same as theirs. Additionally, CHK1 is not the only target to be suppressed by the CHK1 inhibitors they used. So, in fact, this difference does not constitute a conflict. Interestingly, it also provides a valuable indication that many other molecules correlate with the role of CHK1. Therefore, we will conduct further research into the relationship between CHK1 and the inherent properties of ER/PR status, as well as into other molecules related to tumor heterogeneity in breast cancer.

In summary, we first demonstrated the roles of CHK1 related to ER/PR status in aspects of both phenotype and mechanism. CHK1 acts two distinct roles; one is characterized by conditional induction, and the other is essential for cell survival. And the conversion of CHK1'role mainly depends on ER/PR status which determines that CHK1 inhibition is a sensitizer for ADR toxicity in ER^-^/PR^-^/HER2^-^ breast cancer and an independent damage factor in ER^+^/PR^+^/HER2^-^ breast cancer. Moreover, our findings indicate that the cell cycle arrest contributes to the repair for tumor cells exposed to ADR toxicity, but it is also present in the anti-tumor effect caused by CHK1 inhibition. The two-faced role of cell cycle arrest inspires us to further consider the effects of CHK1 on the cell cycle arrest and ultimate fate of cells. We demonstrate that cell cycle arrest itself does not directly determine the ultimate fate of cancer cells but is a reactive activity in response to genomic defects. Therefore, we argue that accurate localization of cell cycle regulation in maintaining cell survival is a prerequisite for rational use of CHK1-targeted intervention.

## Supplementary Material

Supplementary figures and tables.
