# The Role of CHK1 Varies with the Status of Oestrogen-receptor and Progesterone-receptor in the Targeted Therapy for Breast Cancer : Erratum

**DOI:** 10.7150/ijbs.58372

**Published:** 2021-02-20

**Authors:** Wei Xu, Minghua Huang, Jia Guo, Huiting Zhang, Depeng Wang, Tiantian Liu, Haiting Liu, Shiming Chen, Peng Gao, Kun Mu

**Affiliations:** 1 Department of Pathology, School of Basic Medical Sciences, Shandong University, Jinan, 250012, China.; 2 Department of Respiratory and Critical Care Medicine, The second affiliated Hospital of Nanchang University, Nanchang, 330006, China.; 3 Department of Pathology, Qilu Hospital, Shandong University, Jinan, 250012, China.

Recently, we have found a mistake in Figure 3I of our paper^1^. In the original manuscript submitted to reviewers, the content of Figure 3I is correct. However, in the process of marking the molecular weight of the indicated proteins according to reviewers' comments, the content of the Figure 3I was completely covered by Figure 3F due to our operational mistake. It should be emphasized that this mistake occurred in the typesetting process of Figure 3 and did not affect the research results and conclusion of this article. Here, Figure 3 has been presented as follow with the corrected Figure 3I, and there is no need to change the legend. All authors have agreed to the Erratum, and we apologize for the negligence in our work and hope to get the opportunity to correct this mistake.

## Figures and Tables

**Figure 3:**
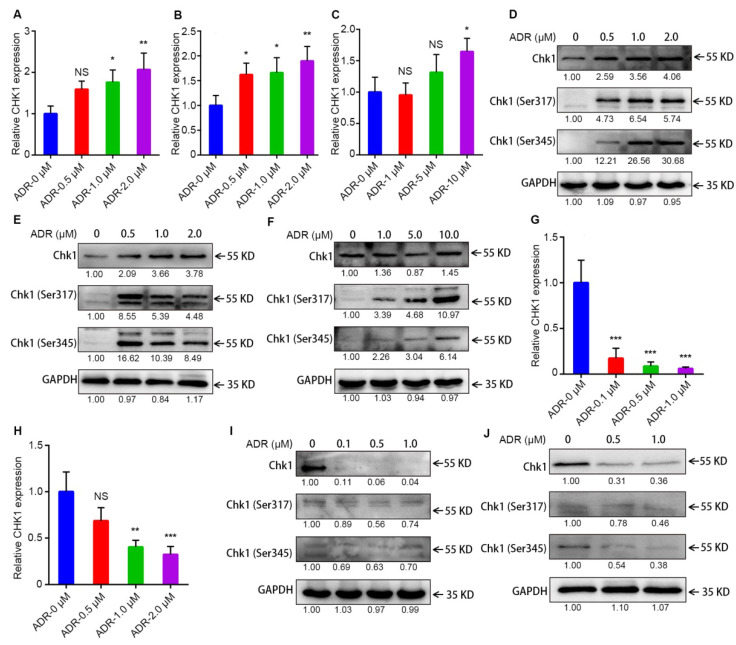
** Activation of CHK1 by ADR depends on ER/PR status. A-C, G-H** The mRNA level of CHK1 detected by RT-qPCR in MDA-MB-231 (**A**), MDA-MB-468 (**B**), MDA-MB-231/ADR (**C**), MCF-7 (**G**) and T47D (**H**) cells, with or without ADR (48 h; 0.5, 1, 2, 5 and 10 μM). **D-F, I-J** Protein levels of CHK1 in MDA-MB-231 (**D**), MDA-MB-468 (**E**), MDA-MB-231/ADR (**F**), MCF-7 (**I**) and T47D (**J**) cells, with or without ADR (48 h; 0.5, 1, 2, 5 and 10 μM), detected by Western blot. Data shown represent the means (± SD) of three independent experiments; *P < 0.05, **P < 0.01, ***P < 0.001; NS, not significant; one-way ANOVA (**A-C, G-H**).
