# Supplementary material for: The Role of CHK1 Varies with the Status of Oestrogen-receptor and Progesterone-receptor in the Targeted Therapy for Breast Cancer
Source: Int J Biol Sci. 2020 Feb 21;16(8):1388–402. doi: 10.7150/ijbs.41627 (PMC7085233; doi:10.7150/ijbs.41627)
Supplement: Supplementary file 1 — Supplementary figures and tables. [file ijbsv16p1388s1.pdf]

Figure S1

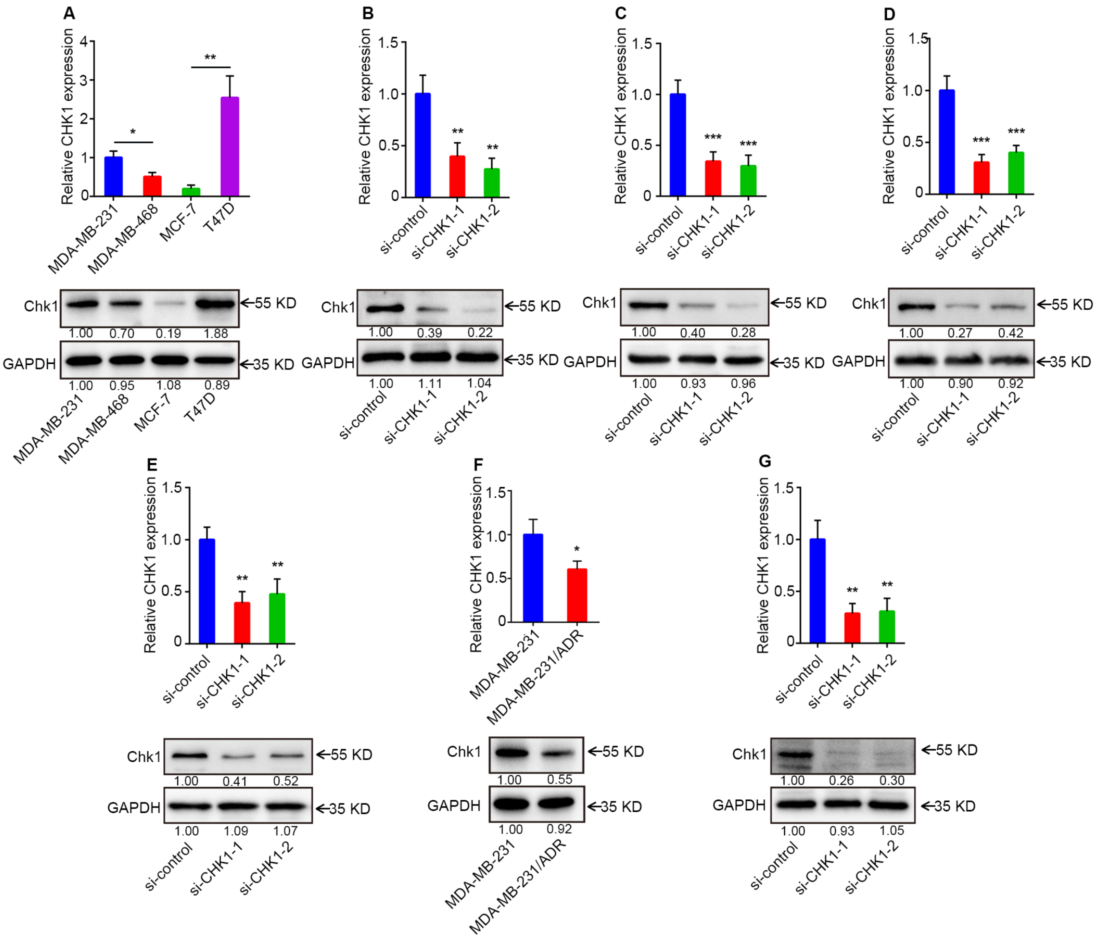

Figure S1 Endogenic expression and knockdown efficiency of CHK1 in the five cell lines. **A** Endogenic expression of CHK1 measured by RT-qPCR and Western blot. There were a low-CHK1 expression and a high-CHK1 expression cell line for each type of cancer cell. **B-E** The knockdown efficiency of CHK1 was detected 48 h after transfection by RT-qPCR and Western blot. both CHK1-siRNA-1 and CHK1-siRNA-2 caused a significant reduction of CHK1 mRNA and protein in MDA-MB-231 (**B**), MDA-MB-468 (**C**), MCF-7 (**D**) and T47D (**E**). **F** RT-qPCR and Western blot showed that the expression of CHK1 decreased in MDA-MB-231/ADR cells compared with MDA-MB-231 cells. **G** The knockdown efficiency of CHK1 in MDA-MB-231/ADR cells. Data shown represent the means (± SD) of three independent experiments; **P* < 0.05, ***P* < 0.01, ****P* < 0.001; one-way ANOVA (**B-E, G**), Student's *t* test (**A, F**).

Figure S2

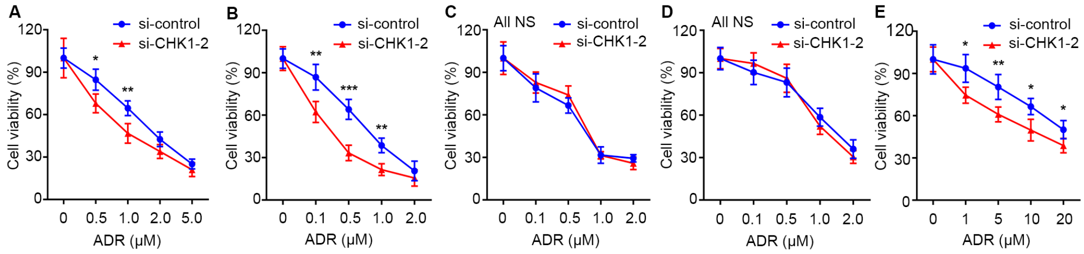

Figure S2 The effects of CHK1 inhibition (CHK1-siRNA-2) on ADR sensitivity in MDA-MB-231 (**A**), MDA-MB-468 (**B**), MCF-7 (**C**), T47D (**D**) and MDA-MB-231/ADR (**E**) cells. Data shown represent the means (± SD) of three independent experiments; **P* < 0.05, ***P* < 0.01, ****P* < 0.001; NS, not significant; Student's *t* test (**A-E**).

Figure S3

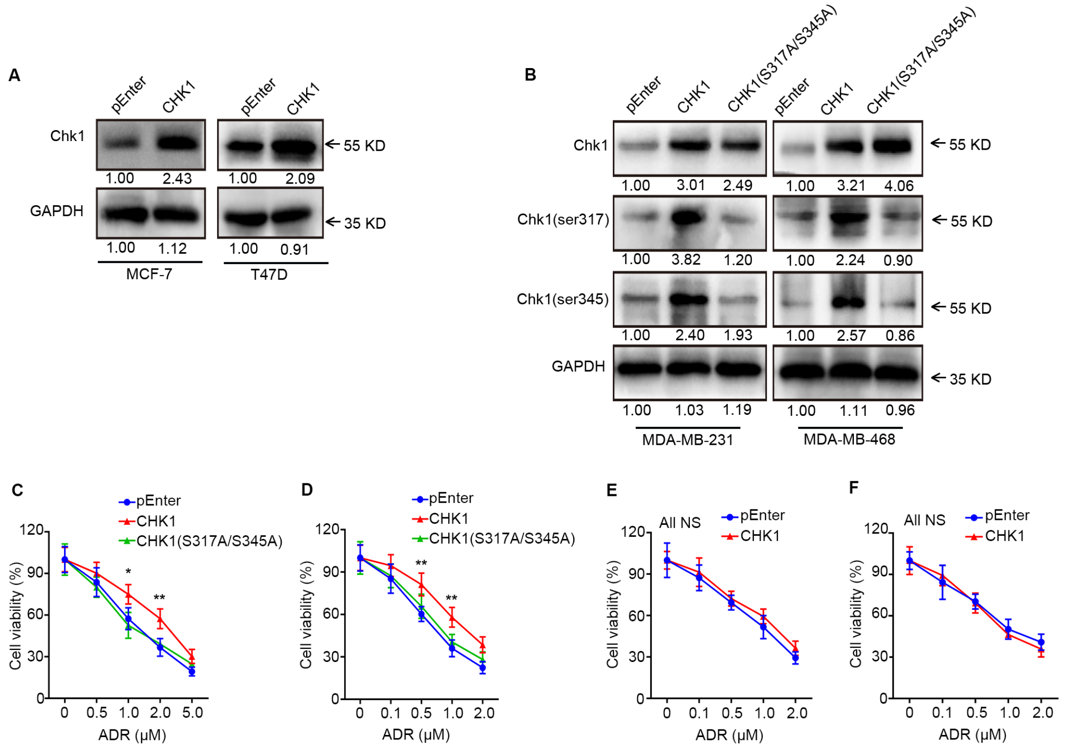

Figure S3 The effects of CHK1 or its kinase dead mutant on ADR sensitivity. **A** The overexpression efficiency of CHK1 was measured by Western Blot in MCF-7 and T47D. **B** The overexpression efficiency and phosphorylation of CHK1 was measured by Western Blot in MDA-MB-231 and MDA-MB-468. C-F The effects of CHK1 or its kinase dead mutant on ADR sensitivity in MDA-MB-231, MDA-MB-468, MCF-7 and T47D. Data shown represent the means (± SD) of three independent experiments; *$P < 0.05$, **$P < 0.01$; NS, not significant; one-way ANOVA (**C, D**) or Student's t test (**E, F**).

Figure S4

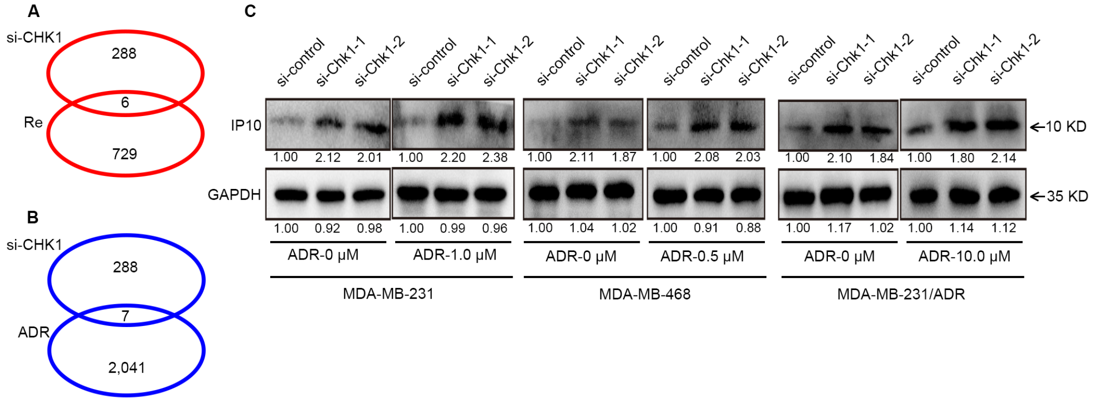

Figure S4 Analysis and verification of the differentially expressed mRNA of the si-CHK1 and si-control groups in MDA-MB-231 cells. **A-B** We intersected differentially expressed genes of the si-CHK1 and si-control groups in MDA-MB-231 cells with genes that had expression variance between ADR-resistant strains and ADR-susceptible strains from GSE24460 (**A**) or the ADR-treated group and the drug-free group in MDA-MB-231 cells from GSE116441 (**B**). **C** Western blot results showed that CHK1-targeted inhibition increased IP10 protein levels with or without ADR in $ER^{-}/PR^{-}/HER2^{-}$ cancer cells.

Figure S5

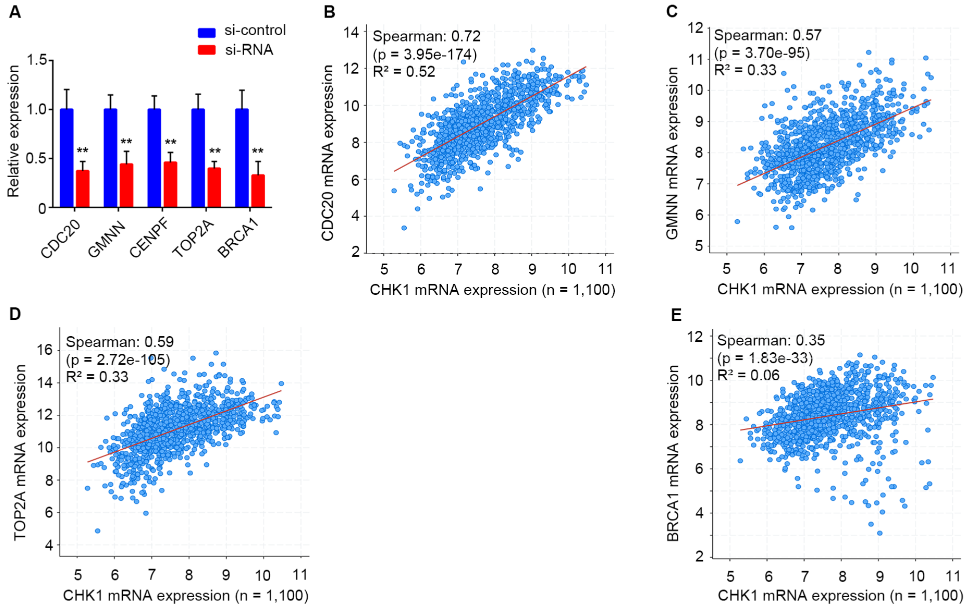

Figure S5 A Knockdown efficiency of CDC20, GMNN, CENPF, TOP2A and BRCA1 in MCF-7 cells. Data shown represent the means (± SD) of three independent experiments; ***$P < 0.01$; Student's *t* test. **B-E** We obtained correlation analysis of CHK1 and CDC20, GMNN, TOP2A, or BRCA1 from the TCGA database using the linear-regression model in cBioPortal.

Figure S6

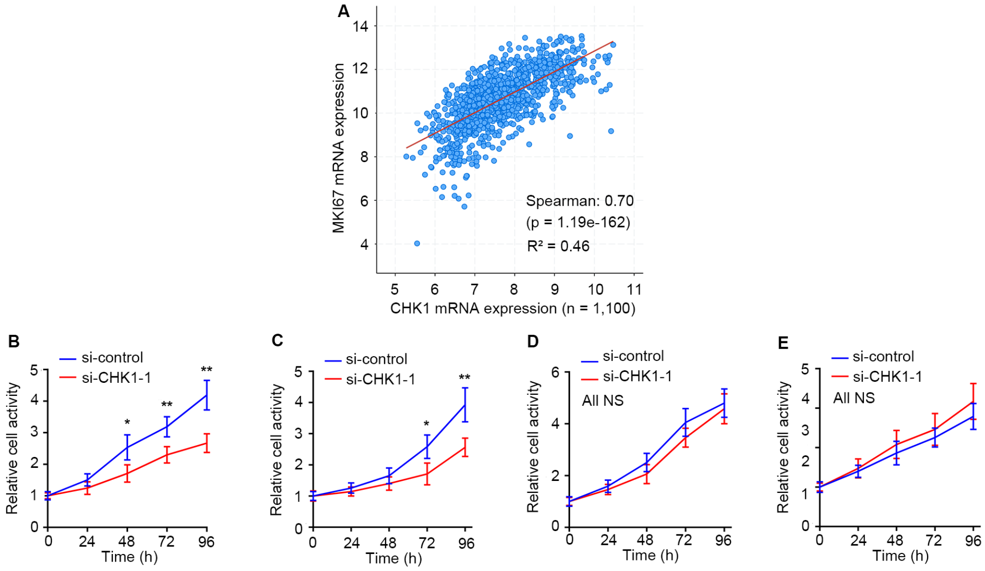

Figure S6 Effects of CHK1 on cell proliferation and recurrence-free survival in breast cancer. **A** Correlation analysis of CHK1 and MKI67 from the TCGA database using cBioPortal. **B-E** We evaluated cell proliferation via CCK-8 assay. CHK1 knockdown (CHK1-siRNA-2) inhibited cell proliferation activities in MCF-7 (**B**) and T47D (**C**) cells but not in MDA-MB-231 (**D**) or MDA-MB-468 (**E**) cells. Data shown represent the means (± SD) of three independent experiments; *$P < 0.05$, **$P < 0.01$; NS, not significant; linear regression (**A**) or Student's *t* test (**B-E**)

Figure S7

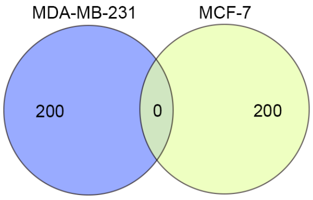

Figure S7 No intersection in the top 200 of differentially expressed genes between the si-CHK1 and si-control groups ranked by multiple in MCF-7 (log2 [fold change] > 0.5 or < −0.5, P < 0.05) or MDA-MB-231(old change > 2 or < 0.5, P < 0.10).

Table S1 Sequences of siRNAs.

Sequences of siRNA	Sequence (5'→3')
si-control	UUCUCCGAACGUGUCACGUTT
CHK1-siRNA-1	GCAGACAAAUCUUAUCAAUGC
CHK1-siRNA-2	GUGGUUUAUCUGCAUGGUAUU
CENPF-siRNA-1	CCCAAGAGAAUGGGACUCUUA
CENPF-siRNA-2	GCGAGUCAGAUCAAGGAGAAU
GMNN-siRNA	CUACGGAUGCAAAGCCAUGUA
TOP2A-siRNA	GCCUGAUUUGUCUAAGUUUAA
CDC20-siRNA	UGGUGGUAAUGAUAACUUGGU
BRCA1-siRNA	CAGCAGUUUAUUACUCACUAAATT

Table S2 List of primers used for quantitative real time-PCR.

Gene Symbol	Sequence (5’→3’)
GAPDH forward	GCACCGTCAAGGCTGAGAAC
GAPDH reverse	TGGTGAAGACGCCAGTGGA
CHK1 forward	TCCGGCTTTCTAAGGGTGATG
CHK1 reverse	TGCTCACAATATCAATCAGCTTCC
CDC20 forward	CTTCCCTGCCAGACCGTATC
CDC20 reverse	AGGATGTCACCAGAGCTTGC
GMNN forward	GCAGGCTTGTCCAAAAGGAA
GMNN reverse	TGACTCCTGGGTGACTCCTC
GENPF forward	GACCAGGCGTCAACCAAGTA
GENPF reverse	GGCCTTCATCTGGATGCACT
TOP2A forward	TCTCAGAGCTTCCCGTCAGA
TOP2A reverse	GGTGTCTTCTCGGTGCCATT
BRCA1 forward	AGCAGCGGATACAACCTCAA
BRCA1 reverse	CCCACACTGCAATAAGTTGCC
